# Non-Pharmacological Interventions for Reducing Anxiety in Patients with Potentially Malignant Oral Disorders

**DOI:** 10.3390/jcm9030622

**Published:** 2020-02-26

**Authors:** Elia Lopez-Yufera, Pia López-Jornet, Oscar Toralla, Eduardo Pons-Fuster López

**Affiliations:** 1 Department Stomatology School of Medicine, University of Murcia, Adv Marques de los Velez s/n, Murcia 30008, Spain; jmpfo@gmail.com; 2 Biomedical Research Institute (IMIB-Arrixaca) School of Medicine, University of Murcia, Adv Marques de los Velez s/n, Murcia 30008, Spain; eduardo.p.f@um.es; 3 Colaborate Department Stomatology School of Medicine, University of Murcia, Adv Marques de los Velez s/n, Murcia 30008, Spain; otoralla@gmail.com

**Keywords:** Anxiety, music therapy, oral disease

## Abstract

Objective: To evaluate the effect of a music intervention upon anxiety, blood pressure, and heart rate in adult patients with potentially malignant oral disorders. Methods: Eighty consecutive adults (mean age: 68.3 years) consulting a Unit of Oral Medicine (Murcia Spain) were randomized to a study group (*n* = 40) that listened to music through headphones during 10 min or to a control group (*n* = 40). Corah’s dental anxiety score, blood pressure, heart rate, oxygen saturation (oximetry), skin temperature, and salivation were recorded at different timepoints before and after patient consultation. Results: Significant pre- versus post-consultation reductions were observed in blood pressure (*p* < 0.001) and heart rate (*p* < 0.001), though not in temperature, salivation, and oxygen saturation (*p* > 0.05). There were no significant differences between the study group and the controls (*p* > 0.05). Conclusions: The applied music intervention had no apparent effect upon anxiety. Further studies using different music intervention strategies and/or analytic parameters are needed to explore the benefits of this approach to decreasing anxiety.

## 1. Introduction 

Oral cancer is associated with smoking and alcohol consumption, among other risk factors. Early detection of potentially malignant disorders is extremely important to detect oral malignancies in the early stages. Different studies have shown cancer of the oral cavity to be characterized by the existence of precursor lesions that are associated with an increased probability of progression towards oral squamous cell carcinoma [1]. Such precursor lesions are referred to as potentially malignant disorders of the oral cavity (PMDs). Systematic reviews have found progression of these lesions towards cancer to occur in an average of 3.5% of all cases. The variables with the greatest influence upon malignization include female gender, combined lesions such as leukoplakia–erythroplakia, lesion surfaces in excess of 200 mm^2^, advanced age, and high degrees of dysplasia [1,2,3]. Patients with such lesions therefore need to undergo periodic evaluation.

A number of options are available for the management of anxiety, including explanation of the treatment process to the patient, medications strategies, biofeedback, hypnosis, and behavioral interventions [4,5,6]. The administration of sedatives and other drugs to lessen patient anxiety before surgery is a common practice, though they often have negative side effects and can prolong patient recovery [6,7,8]. As a result, greater attention currently focuses on the use of non-pharmacological strategies to reduce preoperative anxiety, such as music therapies and interventions. Music is believed to lessen anxiety thanks to its relaxing or distracting effects, which in turn reduce the activity of the neuroendocrine and sympathetic nervous systems [8,9,10,11].

Anxiety is a complex and multifactorial phenomenon that often causes people to avoid seeking dental treatment, and this in turn can have a significant negative impact upon oral health. Intense pre-treatment anxiety can increase pain sensation and hinder or slow optimum recovery, giving rise to negative physiological manifestations, slower wound healing, and increased infection risk [4,7]. Likewise, anxiety can complicate the induction of anesthesia and postoperative recovery [3,12,13,14,15,16]. The literature describes a high demand for sedation during surgery, since anxiety and stress generate fear in patients [17].

Music and music therapy can afford direct physiological, psychological, and social-emotional benefits for patients. The relationship between music and medicine has been widely investigated. Steelman found music interventions to be associated with lowered blood pressure in patients subjected to local anesthesia [18]. In turn, Marwick reported that music therapy could induce patient relaxation and reduce blood pressure and heart rate [19]. Music interventions offer the added benefit of lessening patient exposure to worrisome sounds during treatment [20]. Such interventions affect not only patient´s physiological functions such as blood pressure and heart rate, but also emotions. Music also appears to be a common and effective tool for lessening anxiety in everyday life [4,17,18,19,20,21,22,23,24].

Music therapy has been used as an alternative for reducing patient anxiety in different fields such as psychology, medicine, and dentistry [4,12]. Music not only has an emotional impact but also acts directly upon the sympathetic nervous system, reducing its activity [6,9]. As a result, patients experience psychological as well as physiological benefits such as lowered blood pressure, heart rate, and respiratory frequency [9,16,17,18,22].

Our working hypothesis was that music intervention would have positive effects upon patients’ vital signs. In this regard, to the best of our knowledge, no previous studies have measured the anxiety-lowering potential of music interventions in patients consulting due to potentially malignant disorders of the oral cavity.

The present study was carried out to evaluate the effect of a music intervention upon anxiety, hemodynamic parameters, oxygen saturation (SaO_2_), temperature, and salivation in adult patients before and after consultation in a Unit of Oral Medicine due to potentially malignant oral disorders

## 2. Material and Methods

### 2.1. Patients

A randomized clinical trial was carried out involving two patient groups: study and control. The patients were consecutively recruited in the Unit of Oral Medicine. The initial sample consisted of 90 eligible patients; 10 of them failed to sign the informed consent document and were excluded from the study, thus leaving a final study sample of 80 patients. The study was randomized, experimental, and prospective

All the patients were seen in the Unit of Oral Medicine for the scheduled control of PMD of the oral cavity (leukoplakia and oral lichen planus, lupus erythematosus, actinic keratosis). The inclusion criteria were patients over 65 years of age, adequate capacity to follow instructions and answer questions, and the presence of an oral mucosal lesion compatible with PMD. The exclusion criteria were patients receiving treatment for anxiety, any severe and uncontrolled pain, and the presence of decompensated systemic disease.

The study was carried out following the guidelines of the Declaration of Helsinki, and the trial protocol was approved by the Ethics Committee of the University of Murcia (ID: 1737/2018)

### 2.2. Procedure

Patients were taken to a waiting room where they received a complete description of the study. All patients were informed about the purpose and procedures of the study and gave their written consent to participation in the trial. A trained investigator (ELY) collected the study information. A computer-generated randomization list was used to assign the patients to the study group (listening to relaxing music with headphones from an MP3 player and with access to the volume control) or to the control group (resting in silence with headphones on but without music). The patients listened to different tracks from the sample playlist. Both groups underwent this procedure during 10 min and before their medical intervention. 

Subsequently, patients entered the dentist office where their oral lesions were checked. The different parameters of the study were measured two times: once before they listened to the music, and the second after the visit to the dentist office. 

### 2.3. Psychometric Materials

Data referred to patient age and gender were collected, as well as those regarding habits (tobacco and alcohol use) and sociocultural attributes. Corah’s dental anxiety validated scale was applied [25]. This instrument comprises four questions with 5 possible responses scored from 1 to 5 (where 1 = calm state, and 5 = intense anxiety). The total score is obtained as the sum of the scores corresponding to each of the four questions (range 0–20 points). Four patient anxiety levels were established based on the results of the Corah’s dental anxiety scale: no anxiety (score <4 points), mild anxiety (4–8 points), moderate anxiety (9–12 points), and severe anxiety (>12 points).

The anxiety symptoms were evaluated by means of the Hospital Anxiety and Depression validated Scale (HADS) [26], which comprises 14 items and provides a total score and separate sub-scores for anxiety and depression.

### 2.4. Sialometry

In order to avoid possible contamination from other sources, the patients were instructed to rinse the mouth thoroughly before saliva sample collection. The subjects were required to avoid physical exercise, smoking, eating for two hours before sampling. Unstimulated whole saliva was collected before and immediately after consultation by passive drooling using the draining method (mL/5 min) described by Navazesh and Christensen [27], always during the morning (9–12 h). 

Hemodynamic parameters were determined in the form of blood pressure and heart rate using an automatic digital device (HEM-RML·31, OMRON Healthcare, Kyoto, Japan) according to the instructions of the manufacturer, together with oximetry (MD300C11 pulse oximeter, China) and the recording of skin temperature. The measurements were carried out at two different timepoints: pre- and post-consultation. 

### 2.5. Statistical Analysis

A basic descriptive analysis was made of the study data. Comparisons between groups were established using the chi-squared test in the case of qualitative variables, while in the case of quantitative variables, the Student *t*-test was used to compare means between groups, after confirming a normal data distribution with the Shapiro–Wilk test and checking the homogeneity of variances with the Levene test. Two-factor repeated measures analysis of variance (ANOVA) was performed to determine whether the changes in the scores over time were dependent upon pain, gender, or age. SPSS software (version 23.0, SPSS Inc., Chicago, IL, USA) Statistical significance was considered for *p* < 0.05.

### 2.6. Ethical Approval

All procedures were in accordance with the ethical standards of the institutional and/or national research committee and with the 1964 Helsinki declaration and its later amendments or comparable ethical standards for studies involving human participants. ID: 1737/2018. Informed consent was obtained from all individual participants included in the study.

## 3. Results

The final study sample consisted of 80 patients (24 males and 56 females) with a mean age of 68.3 ± 2.8 years (range 65–76 years). Table 1 shows the descriptive and comparative findings referred to the demographic variables and habits of the patients according to the group (control or study). Table 2 shows that there were no statistically significant differences between the groups. The latter were therefore homogeneous with regard to these variables, and group bias consequently could be ruled out.

Table 3 shows a significant decrease in blood pressure (*p* < 0.001) and heart rate after patient consultation (*p* < 0.001). In contrast, there were no significant differences in oxygen saturation, temperature, salivation, or Corah’s dental anxiety score (*p* > 0.05 in all cases). There was no significant interaction between patient group and time, thus indicating that the passing of time influenced both groups similarly and evidencing that the music intervention did not result in any post- versus pre-intervention increase or decrease in the study parameters (*p* > 0.05).

## 4. Discussion

The present study recorded no significant effects of the music intervention upon patient anxiety, though there were very significant reductions in hemodynamic parameters (blood pressure and heart rate) at the end of the study versus the baseline values. There is abundant evidence of the efficacy of music interventions in reducing patient anxiety in different medical scenarios. [10,11] In this regard, systematic Cochrane reviews have shown music to have a moderate-to-strong impact upon anxiety in cancer patients, patients with mechanical ventilation problems, and individuals with coronary disease [10,11,12,13]. Listening to music has also been found to be effective in reducing anticipatory anxiety in acutely stressful situations.

PMDs of the oral cavity comprise a series of conditions with different malignization potentials, fundamentally attributable to patient toxic habits in some cases and to the inherent characteristics of the disorder in others [1,2,3]. A PMD follow-up algorithm is needed to personalize patient monitoring in the presence of risk factors. It is therefore very important for patients to undergo periodic control visits. 

Decrease in dental anxiety at the end of the intervention versus baseline anxiety was seen in both the study group and the controls. As a result, the decrease cannot be attributed to the music intervention, in contrast to the observations of other studies [20] and systematic reviews [8], where musical distraction has been found to offer benefits in this regard. One possible explanation for the lack of effect of the music intervention upon dental anxiety may be the fact that most of the patients presented only moderate anxiety at the start of the intervention, with an initial Corah’s dental anxiety score of 12, which made it difficult for the anxiety levels to decrease any further. If more anxious patients were analyzed, the intervention might exert a significant effect.

In 2010, Tran et al. examined the anxiolytic intervention preferences of dentists and found soothing background music to be the most widely adopted strategy (83%) in dental treatments [22]. In our study, the investigator selected the music to be listened to by the patients through headphones during 10 min before the intervention.

In 2011, Kim et al. reported that in a study of 219 patients subjected to the surgical removal of an impacted lower third molar, listening to music selected by the patients significantly reduced intraoperative anxiety compared with a group of controls not listening to music [20]. 

In 2015, Thoma et al. randomized 92 adults scheduled for dental treatment to either a music intervention group or a control group. Participants in the music group listened to 10 min of Latin choral music (Miserere by Allegri). Control group participants sat in silence for 10 min, wearing headphones prior to dental treatment. Listening to music proved effective in reducing self-reported anxiety, though no improvements in terms of stress or mood state were observed [17]. In this regard, being able to choose the music may give the patient a certain sense of control of the situation, and this is important for dealing with pain and anxiety. Self-selection of music may also result in a less threatening environment, with increased patient comfort and calmness, both while waiting and during actual dental treatment [4,5,6,7].

A number of individual factors influence patient response to music, including age, gender, cognitive function, the intensity of anxiety, familiarity with and preferences for music, cultural aspects, and personal associations with music [4,6,20]. Because of these individual factors, it is clear that a concrete type of music cannot be universally prescribed for the reduction of stress [5,6,7,8,9,10,11,12,13].

Our study has a number of limitations. On one hand, patients’ musical preferences were not taken into account, and on the other the initial anxiety level was moderate. Another consideration is that the intervention was performed in patients older than 65 years where such oral lesions are more prevalent. It is important to identify those patient subgroups that may benefit from music in the context of potentially malignant disorders of the oral cavity, since this intervention may serve as a complement to dental care and help improve adherence to therapy. It is important to mention that music intervention does not constitute a systematic treatment process, in contrast to music therapy, which is a systematized and individualized process implemented by a professional [5,11]. 

## 5. Conclusions

In conclusion, the music intervention with headphones in the dental office was associated with a very significant decrease in blood pressure and heart rate but had no apparent effect upon anxiety. Further studies are needed (larger samples, objective measures of analytical parameters such as salivary cortisol, α-amylase, electromyogram) to explore strategies for lessening patient anxiety. 

## Figures and Tables

**Table 1:** Comparison of demographic characteristics and habits of the intervention and control groups.

Variable	Group		Test	*p*-Value
	Control	Study		
Age, mean (SD)	67.3 ± 1.2	68.1 ± 1.3	t(78) = −1.021	0.310
Gender, *n* (%)			χ2(1) = 0.238	0.626
Male	11 (27.5)	13 (32.5)		
Female	29 (72.5)	27 (67.5)		
Educational level, *n* (%)			χ2(3) = 0.555	0.907
None	22 (55)	20 (50)		
Basic	8 (20)	7 (17.5)		
Secondary	7 (17.5)	9 (22.5)		
University	3 (7.5)	4 (10)		
Alcohol, *n* (%)			χ2(3) = 1.207	0.751
No	32 (80)	30 (75)		
1–2 times/week	3 (7.5)	4 (10)		
Weekends	0 (0)	1 (2.5)		
Daily	5 (12.5)	5 (12.5)		
Smoking, *n* (%)			χ2(2) = 0.467	0.792
No	29 (72.5)	31 (77.5)		
Yes	5 (12.5)	5 (12.5)		
Ex-smoker	6 (15)	4 (10)		
Dental trauma experience, *n* (%)			χ2(1) = 0.238	0.626
No	27 (67.5)	29 (72.5)		
Yes	13 (32.5)	11 (27.5)		

**Table 2:** Comparison of baseline (pre-consultation) measures between intervention and control groups.

Variable	Group		Student *t*-test	
	Control	Study	t(78)	*p*-Value
Anxiety (HADS)	8.3 (3.8)	7.2 (3.4)	1.402	0.165
Depression (HADS)	5.2 (3.4)	4.7 (2.6)	0.765	0.447
Systolic blood pressure (mmHg)	150.8 (22.53)	155.2 (23.27)	−0.859	1.314
Diastolic blood pressure (mmHg)	80.35 (11.7)	80.45 (7.45)	−0.046	0.393
Heart rate (bpm)	77.6 (14.82)	80.25 (13.9)	−0.825	0.964
SaO_2_ (%)	96.87 (1.22)	97.25 (0.67)	−1.7	0.412
Skin temperature (°C)	35.68 (0.61)	35.74 (0.52)	−0.49	0.093
Salivation (mL/5 min)	1.26 (0.7)	1.44 (0.66)	−1.213	0.625
Corah’s dental anxiety score	11.43 (4.87)	12.05 (4.04)	−0.625	0.504

Hospital Anxiety and Depression Scale (HADS).

**Table 3:** Comparison of pre- and post-consultation changes in the study measures between intervention and control groups.

Variable	Value		Within-Subject Effects	
	Pre- Mean (SD)	Post- Mean (SD)	Time	Group*Time
			F (df); *p*-Value (eta2)	F (df); *p*-Value (eta2)
Systolic blood pressure (mmHg)			F(1.78) = 213.09; *p* < **0.001** (0.732)	F(1.78) = 2.119; *p* = 0.149 (0.038)
Without music	150.80 (22.5)	145.33 (21.7)		
With music	155.20 (23.3)	145.37 (20.0)		
Total	153.00 (22.9)	145.35 (20.7)		
Diastolic blood pressure (mmHg)			F(1.78) = 171.34; *p* < **0.001** (0.687)	F(1.78) = 3.107; *p* = 0.082 (0.041)
Without music	80.35 (11.7)	76.40 (10.7)		
With music	80.45 (7.4)	73.80 (6.1)		
Total	80.40 (9.7)	75.10 (8.8)		
Heart rate (bpm)			F(1.78) = 142.35; *p* < **0.001** (0.646)	F(1.78) = 2.928; *p* = 0.910 (0.004)
Without music	77.60 (14.8)	72.60 (12.4)		
With music	80.25 (13.9)	72.10 (10.7)		
Total	78.92 (14.3)	72.35 (11.5)		
SaO_2_ (%)			F(1.78) = 2.178; *p* = 0.144 (0.034)	F(1.78) = 3.145; *p* = 0.080 (0.041)
Without music	96.87 (1.2)	97.28 (1.1)		
With music	97.25 (0.7)	98.05 (0.6)		
Total	97.06 (1.0)	97.66 (1.0)		
Temperature (°C)			F(1.78) = 1.587; *p* = 0.211 (0.030)	F(1.78) = 0.43; *p* = 0.513 (0.006)
Without music	35.68 (0.6)	35.60 (0.6)		
With music	35.74 (0.5)	35.68 (0.5)		
Total	35.71 (0.6)	35.64 (0.5)		
Salivation (mL/5 min)			F(1.78) = 1.997; *p* = 0.161 (0.032)	F(1.78) = 3.047; *p* = 0.084 (0.041)
Without music	1.26 (0.7)	1.58 (0.9)		
With music	1.44 (0.7)	2.09 (0.7)		
Total	1.35 (0.7)	1.83 (0.8)		
Corah’s dental anxiety score			F(1.78) = 2.873; *p* = 0.094 (0.041)	F(1.78) = 16.71; *p* = 0.181 (0.031)
Without music	11.43 (4.9)	11.45 (4.9)		
With music	12.05 (4.0)	11.50 (3.9)		
Total	11.74 (4.5)	11.47 (4.4)		

Mean values (standard deviation [SD]).
